# Negative-Pressure Cavitation Extraction of Four Main Vinca Alkaloids from *Catharanthus roseus* Leaves

**DOI:** 10.3390/molecules17088742

**Published:** 2012-07-25

**Authors:** Fansong Mu, Liuqing Yang, Wei Wang, Meng Luo, Yujie Fu, Xiaorui Guo, Yuangang Zu

**Affiliations:** 1 Engineering Research Center of Forestry Bio-preparation, Ministry of Education, Northeast Forestry University, Harbin 150040, China; 2 Key Laboratory of Forest Plant Ecology, Ministry of Education, Northeast Forestry University, Harbin 150040, China; 3 State Engineering Laboratory of Bio-Resource Eco-Utilization, Harbin 150040, China; 4 Zhejiang Hisun Pharmaceutical Co. Ltd., Taizhou 318000, China

**Keywords:** *Catharanthus roseus*, vinca alkaloids, negative-pressure cavitation extraction

## Abstract

In the present study, an improved method termed negative-pressure cavitation extraction (NPCE) followed by reverse phase high-performance liquid chromatography (RP-HPLC) was developed for the extraction and quantification of vindoline (VDL), catharanthine (CTR), vincristine (VCR) and vinblastine (VLB) from *Catharanthus roseus* leaves. The optimized method employed 60-mesh particles, 80% ethanol, a negative pressure of −0.075 MPa, a solid to liquid ratio of 1:20, 30 min of extraction and three extraction cycles. Under these optimized conditions, the extraction yields of VDL, CTR, VCR and VLB are 0.5783, 0.2843, 0.018 and 0.126 mg/g DW, respectively. These extraction yields are equivalent to those from the well-known ultrasonic extraction method and higher than the yields from maceration extraction and heating reflux extraction. Our results suggest that NPCE-RP-HPLC represents an excellent alternative for the extraction and quantification of vinca alkaloids for pilot- and industrial-scale applications.

## 1. Introduction

**Figure 1:**
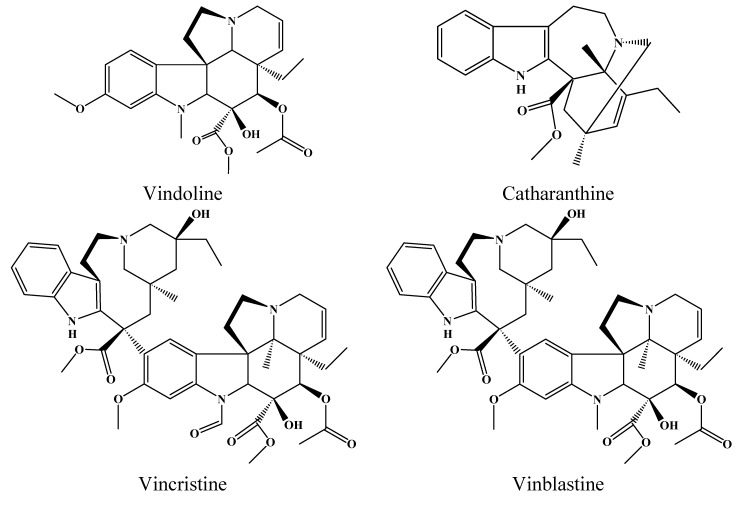
Chemical structure of vindoline (VDL) and catharanthine (CTR), vincristine (VCR), vinblastine (VLB).

*Catharanthus roseus* (L.) G. Don (Madagascar periwinkle) is a terpenoid indole alkaloid (TIA)-producing plant that belongs to the Apocynaceae Family. This plant has historically been used to treat a wide assortment of diseases. Research shows that *Catharanthus roseus (C. roseus)* contains over 130 compounds, and many of which have cytotoxicity [1,2]. Few species have been as thoroughly studied as *C. roseus* and it has become a model species for the study of secondary metabolism in plants. The interest in this species arises from its therapeutic role as the source of the anticancerous alkaloids vinblastine (VLB) and vincristine (VCR) [3,4]. VLB and VCR are bisindole antitumor alkaloids; their monomeric precursor molecules are vindoline (VDL) and catharanthine (CTR) (Figure 1). Vinblastine and vincristine have very similar chemical structures, but their effects on the body are not the same. Vinblastine was introduced in 1960 and is used to treat specific types of cancer including Hodgkin’s disease, breast cancer, testicular cancer and non-small cell lung cancer [5]. Vincristine is an oxidized form of vinblastine and was introduced in 1963. Vincristine is used in the treatment of acute lymphoblastic leukemia (ALL) and has provided a significant advance in successful treatment of ALL in children. When vincristine is added to the treatment regimen for children suffering from ALL, the survival rate reaches eighty percent [6]. The anticancer activity of these alkaloids is attributed to their ability to disrupt microtubules, causing the dissolution of mitotic spindles and metaphase arrest in dividing cells [7,8].

Because they exist in trace amounts (0.01–0.1 mg/g DW) in the plant, the extraction of VLB and VCR and their precursors from the plant has been an important method on the industrial scale. In attempts to improve the production of valuable alkaloids such as VDL, CTR, VCR and VLB, several studies on *C. roseus* have reported the isolation of vinca alkaloids by centrifugal partition chromatography, supercritical CO_2_ extraction, ultrasound-assisted extraction and microwave assisted extraction [9,10,11,12,13]. However, these methods are not suitable for industrial scale production due to the high equipment costs and low material throughput. Therefore, finding high efficiency, low-cost and environmentally friendly methods for the extraction of vinca alkaloids for pilot-scale as well as industrial scale-up production are of great importance.

In recent years, a new extraction method called negative-pressure cavitation extraction (NPCE) has increased in popularity; NPCE is more efficient at constant lower temperatures and intensities than classical extraction technologies such as maceration extraction, heat reflux extraction and ultrasonic assisted extraction. Since it was first developed in 2009, the NPCE technique has been widely used in the extraction of a number of bioactive compounds from Chinese herbs [14,15,16,17,18,19,20]. So far no report has been published on the use of NPCE for alkaloid extraction. In this study, NPCE followed by RP-HPLC detection is proposed for the extraction and quantification of four main vinca alkaloids (VDL, CTR, VCR and VLB) from *C. roseus* leaves. This study evaluated the effects of particle size, solvent concentration, as well as NCPE operational variables including negative pressure, solid to liquid ratio, extraction time and cycles on the extraction yields of the four main vinca alkaloids. This work may provide valuable data for both pilot- and industrial-scale applications.

## 2. Results and Discussion

### 2.1. Effect of Particle Size and Ethanol Concentration

**Figure 2:**
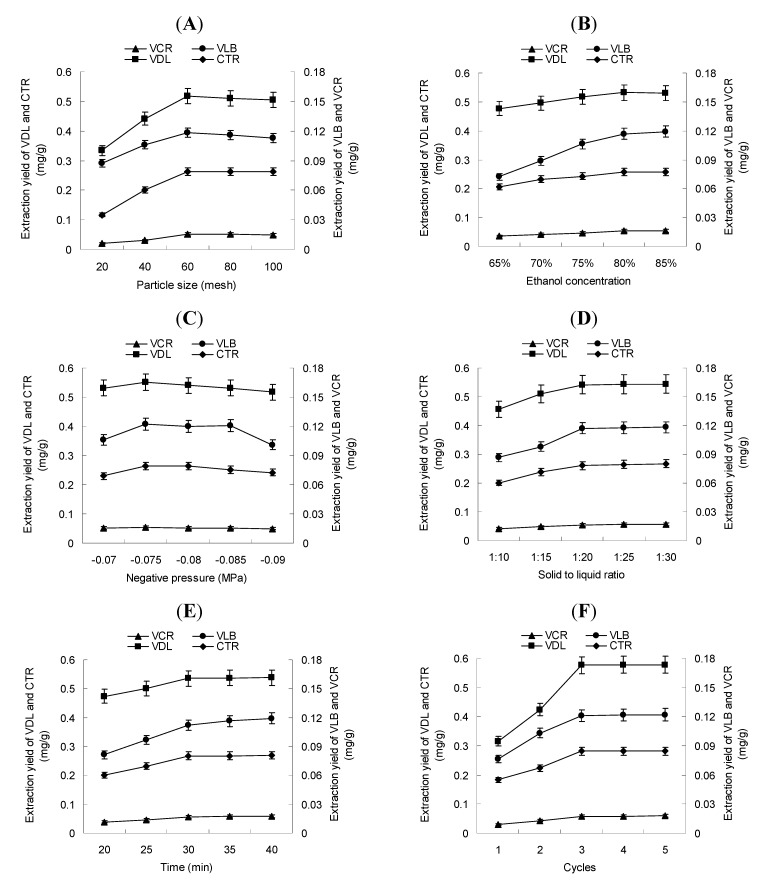
Effect of (**A**) particle size, (**B**) ethanol concentration, (**C**) negative pressure, (**D**) solid to liquid ratio, (**E**) time and (**F**) number of cycles on the yields of VDL, CTR, VCR and VLB of NPCE.

The particle size of the plant material and solvent concentration has a large impact on extraction yields. This is because plant cell walls are broken, and the accessibility to the bioactive compounds is augmented for the extraction solvent. As shown in Figure 2A, the yield of target alkaloids increased significantly when the particle size decreased from 20 to 60 mesh. However, when the particle size ranged from 60 to 100 mesh, the extraction yields decreased slightly. That smaller particles would result in more efficient extraction is not surprising. As the particles become smaller, the diffusion path becomes shorter; consequently, the intra-particle diffusion resistance would be reduced. Smaller particles possess greater surface area, which would cause particles to have more contact with the solvent. Thus, a reduction in mass transfer resistance might facilitate the extraction of bioactive compounds. However, the smallest particles (60–100 mesh) might stay at the surface of the solvent during extraction, limiting their exposition to cavitation and resulting in lower extraction yields. Therefore, a particle size of 60 mesh was selected and used in the following tests.

To increase the extraction efficiency and decrease the extraction time, the extraction solvent concentration was optimized. It is well known that increasing the ethanol concentration induces a reduction in the dielectric constant of the extraction solution and improves the solubility of bioactive compounds. A range of ethanol concentrations (65%, 70%, 75%, 80%, 85%) were compared. As can be seen from Figure 2B, the extraction yields increased until the ethanol concentration reached 80%, with only a slight additional increase between 80% and 85% ethanol. Therefore, taking into account both the extraction yield and the practicality, 80% ethanol was selected for the following experiments. 

### 2.2. Effect of Negative Pressure and Solid to Liquid Ratio

In our experiment, the extraction pressure was adjusted by regulating the airflow. The effect of different extraction pressures on the extraction yields of vinca alkaloids are shown in Figure 2C. A significant effect of the extraction pressure on the extraction yields of vinca alkaloids was observed. The stirring and cavitation effects of NPCE promote extraction of bioactive compounds by means of accelerating the mass transfer and disrupting cells. As shown in Figure 2D, a negative pressure of −0.075 MPa resulted in the highest yield of vinca alkaloids. The cavitation effect is affected intermediately by airflow rate in terms of the negative pressure and amount of cavitation bubbles. It firstly increases rapidly with a decrease in the air flow rate because of the increase of negative pressure and then decreases rapidly as the lack of air results in a decrease in the number of cavitation bubbles. As a result, −0.075 MPa was used to investigate the effects of other parameters.

The effect of the solid to liquid ratio on the extraction yields of the four main vinca alkaloids was determined by a series of experiments with different solid to liquid ratios (1:10, 1:15, 1:20, 1:25 and 1:30, w/v). As is shown in Figure 2D, the extraction efficiency increased with increasing solid to liquid ratios up to 1:20. Less extraction solvent results in the insufficient mixing of sample and solvent. Higher solvent volumes did not significantly improve the extraction efficiency; the turbulent phase may be subject to restriction and destruction resulting in weaker cavitation effects. Considering the extraction yields of the four main vinca alkaloids and the solvent consumption, an optimal solid to liquid ratio of 1:20 was used in the following tests.

### 2.3. Effect of Extraction Time and Extraction Cycle

The proper extraction time is necessary for optimal extraction efficiency and ensuring a distribution equilibrium of bioactive compounds between the plant material and the extraction solvent. In the present study, extraction was carried out for 25, 30, 35, 40 or 45 min and the results are shown in Figure 2E. It is evident that longer extraction times maximized the extraction efficiency of vinca alkaloids in NPCE. The extraction yield of vinca alkaloids dramatically increased with an extension of the extraction time to 30 min. Beyond 30 min, the extraction time had little impact on the extraction yield, indicating that the target molecules reached their distribution equilibrium by 30 min. Given the increase in extraction yield and the low energy consumption, 30 min was selected as the appropriate extraction time and used in the following experiments.

The effect of the number of extraction cycles on the extraction yields was investigated using 1, 2, 3, 4 or 5 cycles. Generally, an increase in the number of extraction cycles increases extraction yield by allowing greater exposure of the material to fresh solvent and favoring the material-solvent equilibrium. As shown in Figure 2F, the extraction yields significantly increased when the number of extraction cycles increased from 1 to 3. After three cycles, the total extraction yields of vinca alkaloids were 97.7, 98.9, 97.6, 96.8 and 98.7%. Hence, three extraction cycles were determined to be efficient extraction of the target compounds.

### 2.4. Comparison of Different Extraction Methods

After optimization, NPCE was shown to be an efficient method with high extraction yields (0.5783 mg/g for VDL, 0.2843 mg/g for CTR, 0.018 mg/g for VCR and 0.126 mg/g for VLB). We determined that the optimal parameters to be employed are: 60-mesh particle size, 80% ethanol, a solid to liquid ratio of 1:20 (w/v) and a 30 min extraction for three cycles. NPCE has received considerable attention for extracting several bioactive compounds from Chinese Herbs; to the best of our knowledge, NPCE of vinca alkaloids has not been reported. As a result, it is necessary to compare the extraction efficiency of NPCE with other extraction methods for the extraction of vinca alkaloids. 

Different methods (ME, HRE, USE and NPCE) were performed using the optimized conditions, and the results are summarized in Table 1. The highest extraction yields of vinca alkaloids were obtained with NPCE. The higher yields obtained with NPCE required a lower temperature, lower energy dissipation and a lower cost relative to ME, HRE and UAE. The extraction yields of vinca alkaloids using NPCE for 30 min at room temperature and USE for 30 min at 45 °C are higher than those using ME for 12 h at room temperature and HRE for 3 h at 80 °C. A comparison of the extraction conditions of the four methods showed that a longer extraction time and more energy consumption were needed for ME and HRE. Furthermore, the extraction efficiency of ME and HRE was the lowest. The comparable extraction efficiency of NPCE and UAE were both a result of the cavitation effect. However, the extraction yields of vinca alkaloids obtained by NPCE were higher than those obtained by UAE. This may be explained as follows. During NPCE, an intense cavitation effect is produced by tiny bubbles. This is followed by a mass transfer effect that includes turbulence, vibration and collision between the extraction solvent and the matrix. The NPCE cavitation effect is more efficient than the acoustic cavitation of UAE that occurs during the extraction process. In addition, the industrial application of ultrasonic cavitation is restricted by its high cost, noise pollution and the degradation of thermo-sensitive compounds at high temperature. Therefore, this investigation shows that NPCE is an efficient and economic method that is suitable for the industrial-scale extraction alkaloids from *C. roseus* leaves.

**Table 1:** Comparison of ME, HRE, UAE and NPCE on the yields of VDL, CTR, VCR and VLB from *C. roseus* leaves. Data are presented as means ± S.D. (*n* = 3).

Yield (mg/g)	ME	HRE	USE	NPCE
VDL	0.5069 ± 0.0242	0.5138 ± 0.0253	0.5767 ± 0.0280	0.5783 ± 0.0278
CTR	0.2271 ± 0.0112	0.2316 ± 0.0115	0.2811 ± 0.0134	0.2843 ± 0.0132
VCR	0.0098 ± 0.0004	0.0116 ± 0.0005	0.0162 ± 0.0006	0.0181 ± 0.0007
VLB	0.0603 ± 0.0028	0.0982 ± 0.0041	0.1140 ± 0.0050	0.1263 ± 0.0058

## 3. Experimental

### 3.1. Plant Material

*C. roseus* leaves (80% water content) were collected from the Greenhouse of Northeast Forestry University, Heilongjiang, China, and identified by Professor Shao-Quan Nie from the Key Laboratory of Forest Plant Ecology, Ministry of Education, Northeast Forestry University, Harbin, China. The material was dried in the shade, powdered by a disintegrator (HX-200A, Yongkang Hardware and Medical Instrument Plant, China) and then sieved through 20–100 mesh sieves (Harbin Ouerfu Filter Material Co., Ltd., China). The powder was kept at 4 °C prior to use.

### 3.2. Chemicals and Reagents

Reference standards of VDL, CTR, VCR and VLB were purchased from Shanghai Anticancer Phytochemistry Co., Ltd. (Shanghai, China). Methanol (Honeywell International Inc., Morristown, NJ, USA) and acetonitrile (Dikma Technologies Inc., Beijing, China) used for HPLC were HPLC reagent grade. Deionized water was filtered using a Milli-Q Ultrapure water purification system from Millipore (Billerica, MA, USA). Other reagents were all commercially available analytical grade chemicals.

### 3.3. Extraction Procedures

#### 3.3.1. Negative Pressure Cavitation Extraction (NPCE)

The apparatus (CN 2597047) used in the NPCE experiment was provided by the Engineering Research Centre of Forestry Bio-preparation, Ministry of Education, Northeast Forestry University, Harbin, China. The mode of action of NPCE is based on the continuous formation and collapse of tiny bubbles under the action of a vacuum, which was described in detail in our previous study [21,22,23]. 

In the present study, 25 g of powdered *C. roseus* leaves were transferred into the extraction chamber with different concentrations of extraction solvent. Then, a vacuum pump was connected to the extraction pot through a condenser to generate negative pressure. The pressure was monitored by adjusting the airflow rate, which was supplied from the bottom of the device. The extraction process was performed under different conditions as described in the Results section. After each extraction, the extraction solutions were combined and dried on a rotary evaporator (RE-52AA, Shanghai Huxi Instrument, Shanghai, China) at 45 °C. HPLC-grade methanol was added in a constant volume to prepare the samples for RP-HPLC analysis. The extraction efficiency of the compounds in a given sample was defined as follows:



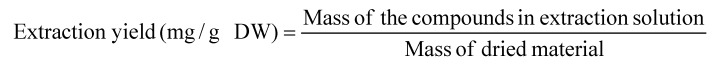



#### 3.3.2. Conventional Extraction Procedures

For maceration extraction (ME), as determined by the preliminary optimized experiments, 25 g of powdered of *C. roseus* leaves were placed in a beaker with 500 mL of 80% aqueous ethanol solution. The beaker was placed at room temperature for 12 h, the filtered extraction solution was collected, and another 500 mL of 80% ethanol was added into the beaker for an additional 12 h to obtain optimal extraction efficiency. 

For heat reflux extraction (HRE), after preliminary optimization experiments, 25 g of powdered *C. roseus* leaves were added to a round-bottom flask with 500 mL of 80% ethanol. The round-bottom flask was then placed into a water bath and linked with a condenser at the joint of the flask. The extraction process was twice maintained at 80 °C for 3 h to obtain optimal extraction efficiency. 

Ultrasonic-assisted extraction (UAE) was run in an ultrasonic bath (Kunshan Ultrasonic Instrument, Kunshan, China) using the conditions optimized for NPCE at normal pressure. In brief, 25 g of powdered of *C. roseus* leaves were added to a triangular flask with 500 mL of 80% ethanol. The extraction was performed in ultrasonic bath at a frequency of 40 kHz with the maximum input power of 250 W at 45 °C for 30 min. Three replicates were performed to obtain the best extraction efficiency. 

### 3.4. RP-HPLC Analysis

In this study, an RP-HPLC separation and analysis protocol is employed to detect the four alkaloids. UV absorbance is the method of choice for their detection, and 220 nm was the wavelength used. The criteria for the identification of the compounds were established based on comparisons of the retention time and spectrum of an unknown compound with HPLC library data of standards [24].

RP-HPLC analysis was carried out using a Jasco HPLC system (Model 1580, Japan), which included a PU-1580 pump, a 7725i manual injector and a UV-1575 detector connected to CkChrom chromatography data system software (Version 6.1). Separations were performed on a HiQ sil C_18_W column (4.6 mm × 250 mm). 

**Figure 3:**
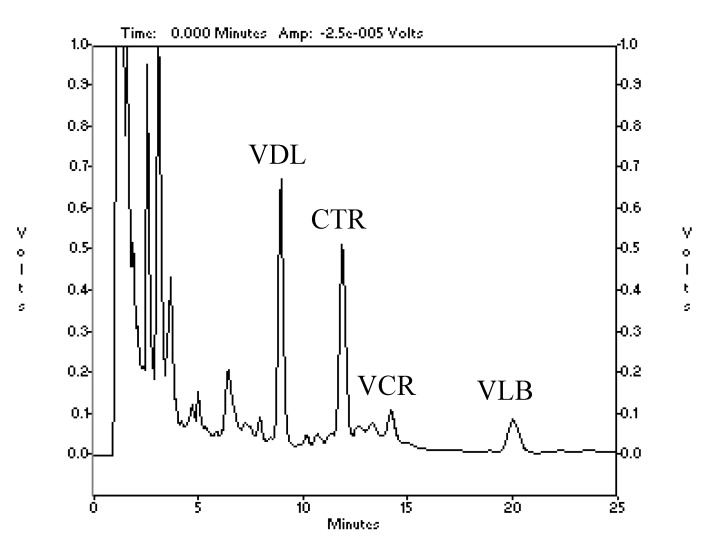
RP-HPLC trace of an extract of *C. roseus* monitored at 220 nm.

The separation was carried out by an isocratic elution using solution A (methanol/acetonitrile = 4:1), and solution B (water/diethylamine = 986:14; pH adjusted to 7.2 by H_3_PO_4_). The mobile phase consisted of 620 mL solution A and 380 mL solution B. The flow rate was set at 2.0 mL·min^−1^ and the elution was monitored at 220 nm [25]. The target alkaloids were resolved sufficiently to give baseline separation within 25 min and the retention times of VDL, CTR, VCR and VLB were 9.0, 11.9, 13.2 and 20.2 min, respectively (Figure 3). Four main vinca alkaloids were identified by comparing their retention times with standard solutions (Table 2). This method is sensitive and accurate with good reproducibility. Validation of the quantification was performed three times. 

**Table 2:** UV absorption and identity of peaks in the chromatogram.

Retention Time (min)	Peak maxima (nm)	Identity	Peak maxima (nm) in literature [ 24]
9.0	213, 251, 305	VDL	214, 254, 306
11.9	223, 283	CTR	226, 282
13.2	220, 256, 297	VCR	222, 256, 298
20.2	215, 265	VLB	214, 266

### 3.5. Statistical Analysis

All experiments were conducted in triplicate，and the average values of extracted vinca alkaloids were used for statistical analysis. The one-way ANOVA test was used to calculate the significance of the differences in extraction efficiency of the target alkaloids. The results of RP-HPLC analysis are expressed as the means ± SD (*n* = 3).

## 4. Conclusions

NPCE was first applied to the extraction of VDL, CTR, VCR and VLB from *C. roseus* leaves and compared with other standard extraction methods. Under the optimal parameters, the extraction yields of VDL, CTR, VCR and VLB were 0.5783, 0.2843, 0.018 and 0.126 mg/g DW, respectively. These yields are comparable to those obtained by the well-known UAE method and significantly higher than those obtained by ME and HRE. The results indicated that NPCE offers a fast, environmentally friendly and straightforward route for vinca alkaloid production. Consequently, the NPCE-RP-HPLC described here represents an excellent alternative for the extraction and quantification of the four main *C. roseus* alkaloids in both pilot- and industrial-scale applications.
